# Sortase A regulates cell wall integrity, quorum sensing, and biofilm formation to modulate adhesion properties in *Lactiplantibacillus plantarum* C8

**DOI:** 10.1128/aem.00029-26

**Published:** 2026-02-25

**Authors:** Youwei Ji, Yiping Yang, Tao Zhang, Xiaoqun Zeng, Weichen Bao, Daodong Pan, Liang Zhao, Huizhen Li, Zhen Wu

**Affiliations:** 1 State Key Laboratory for Quality and Safety of Agro-Products, Zhejiang Key Laboratory of Food Microbiology and Nutritional Health, College of Food Science and Engineering, Ningbo University 47862, Ningbo, Zhejiang, China; 2 Jinhua Yinhe Biological Technology Co., Ltd, Jinhua, Zhejiang, China; 3 College of Food Science and Nutritional Engineering, China Agricultural University 34752, Beijing, China; University of Illinois Urbana-Champaign, Urbana, Illinois, USA

**Keywords:** *Lactobacillus plantarum*, sortase AQ, CRISPR/Cas9, biofilm-quorum sensing, adhesion

## Abstract

*Lactiplantibacillus plantarum,* a ubiquitous probiotic in fermented foods and the human gut, relies on gastrointestinal tract colonization for its health-promoting functions. Central to this colonization is Sortase A (SrtA), a transpeptidase that anchors LPXTG motif-containing proteins to the cell wall peptidoglycan layer. This study investigates the *srtA*-mediated regulatory axis linking cell wall integrity, biofilm formation, and quorum sensing (QS) to adhesion properties in *L. plantarum* C8 (CGMCC No. 30504). Gene Ontology (GO)–KEGG enrichment analysis reveals that *srtA* deletion disrupts pathways critical for environmental adaptation, including two-component signal transduction and AI-2-dependent QS. Furthermore, the results of differential gene expression analysis indicate that *srtA* deletion is associated with the downregulation of genes involved in pyruvate metabolism, amino sugar/nucleotide sugar metabolism (essential for exopolysaccharide biosynthesis), and cell wall-associated signaling cascades, processes linked to adhesion and colonization. The molecular-level alterations were consistent with the observed phenotypic changes, including impaired cell wall integrity, reduced adhesion, and diminished biofilm-forming capacity. These results establish a mechanistic connection between *srtA*-directed cell wall anchoring, QS-regulated biofilm dynamics, and probiotic adhesion efficacy in *L. plantarum*.

**IMPORTANCE:**

Gastrointestinal tract colonization is the foundation of probiotic efficacy, enabling *Lactiplantibacillus plantarum* to modulate the gut microbiota, reinforce intestinal barriers, and confer health benefits. Sortase A (SrtA) is central to this process, covalently anchoring LPXTG-containing surface proteins that mediate adhesion, biofilm formation, and immune modulation. While *srtA*’s role in pathogenic Gram-positive bacteria is well documented, its regulatory functions in non-pathogenic probiotic strains remain largely unexplored—especially regarding its integration with quorum sensing (QS) and environmental adaptation pathways. This study dissects the *srtA*-mediated molecular network in *L. plantarum* C8, revealing s*rtA* as a master regulator integrating cell wall integrity, QS-regulated biofilm dynamics, and surface protein function via pathways including pyruvate and amino sugar/nucleotide sugar metabolism. These insights provide a mechanistic foundation for engineering probiotic strains with enhanced adhesion, colonization, and persistence and offer a scientific basis for developing precision-targeted functional foods and therapeutics.

## INTRODUCTION

*Lactiplantibacillus plantarum,* as an important probiotic, is widely present in a series of fermented food environments (1, 2) and has been proven to survive in gastric transport (3, 4). Its excellent environmental adaptability (5) and potential probiotic functions have made it a hot topic in probiotic research in recent years, and it has been widely used in multiple fields such as food, medicine, and agriculture (6). The realization of these probiotic functions depends on its successful colonization in the host’s intestinal tract, and the adhesion ability of bacteria is one of the key factors affecting their colonization ability (7). However, the colonization ability of *L. plantarum* varies among different strains (8) and is regulated by its surface proteins. Previous studies have shown that *L. plantarum* can effectively regulate the intestinal flora (9), enhance the host’s immune function (10), and inhibit the colonization of pathogenic bacteria (2, 11, 12).

Sortase A (SrtA) is a key enzyme in Gram-positive bacteria. It catalyzes the anchoring of surface proteins to the cell wall by specifically recognizing the LPXTG motif, thereby enhancing the adhesion and colonization ability of bacteria to the host (13–15). In addition, *srtA* mediates the interaction between bacteria and the host immune system by regulating the localization and function of surface proteins (16, 17). Thereby regulating the immune response of the host, it is of great significance to study *srtA* biological functions. Previous studies have proved that *srtA* plays a key role in the infection process of pathogenic bacteria, such as *Staphylococcus aureus* and *Listeria monocytogenes* (18, 19), but the research on its function and probiotic potential in non-pathogenic bacteria, such as *L. plantarum,* is still relatively limited. Current research focuses predominantly on commercial model strains, neglecting potential strain-specific variations in *srtA*-mediated regulation.

With the advancement of gene editing technology, researchers have been able to more accurately analyze the function of *srtA* in different bacteria through means such as gene knock-in and knockout. For instance, Donald et al. studied the mucus-binding protein MUB in *Lactobacillus reuteri* ATCC53608 and found that mutant strains lacking the LPXTG region at the C-terminal were unable to anchor to the cell wall and exhibited significantly reduced mucus binding and aggregation abilities (20). Yamaguchi et al. constructed the *srtA* deletion mutant strain of *Streptococcus hemiformis* using the insertion inactivation strategy (21), confirming that this enzyme plays an important role in the process of bacterial colonization. Furthermore, Ping et al. discovered that curcumin can inhibit the *srtA* activity in *Streptococcus* mutans, thereby reducing the formation of its biofilm (22), providing a new research idea for the treatment of dental caries. These findings emphasize the role of *srtA* as an important factor in bacterial physiological processes and highlight its key position in microbiological research. Nevertheless, *srtA’*s regulatory mechanisms—particularly concerning biofilm formation and quorum sensing (QS) in non-model *L. plantarum* strains—require further elucidation.

Based on the above background, this study intends to take *L. plantarum* C8 (CGMCC No.30504) as the research object and construct *srtA*-deficient lactic acid bacteria strains by using CRISPR/Cas9 gene editing methods. By determining the functional characteristics, such as self-aggregation, hydrophobicity, and cell adhesion of recombinant strains and combining transcriptome data to analyze the changes at the gene level, the mechanism of *srtA* in the adaptive regulation of strains was systematically analyzed. This study aims to deeply understand the mechanism of *srtA* in the colonization and probiotic functions of *L. plantarum*, which supports the development of probiotic preparations and functional foods with LAB fermentation.

## MATERIALS AND METHODS

### Reagents and strains

*L. plantarum* C8 was derived from the China Microbiological Preservation Center (CGMCC No. 30504, China). It was anoxically cultured at 37°C for 24 h in Man Rogosa Sharpe (MRS) medium (Hangzhou, China) and stored in 30% glycerol (−80°C) for future use.

### Extraction of the whole genome of *L. plantarum* C8

The laboratory-preserved strain *L. plantarum* C8 was taken out of the refrigerator at −80°C and inoculated into 100 mL of MRS Liquid medium at an inoculation ratio of 1% for activation. It was then incubated in a constant temperature and humidity incubator (37°C, 8–10 h). The genomic DNA of *L. plantarum* C8 was extracted using the Full Gold Bacteria Genome Extraction Kit. The DNA concentration and purity were verified using an ultramicro spectrophotometer, and then stored in a refrigerator at −20°C for future use.

### Construction of mutant strains with deletion of Sortase A

#### Preparation of *L. plantarum* C8 competence

The preparation steps of *L. plantarum* C8 competence refer to the laboratory of Zhang Feng (23), and some modifications have been made to it. The overnight-activated *L. plantarum* C8 was inoculated into 50 mL of capable medium (containing 6.8332 g sorbitol and 0.5 g glycine) at an inoculation volume of 2%–3%, and then incubated in a constant temperature and humidity incubator at 37°C until OD_600_ = 0.4–0.6. Subsequently, the bacteria were washed 2–3 times with pre-cooled SM buffer (16.3 g sucrose and 16.6618 mg anhydrous magnesium chloride dissolved in 50 mL deionized water) (4°C, 5,000 r/min, 3 min), and the bacteria were resuspended with 2 mL SM buffer. Each tube was divided into 100 μL into 1.5 mL sterile centrifuge tubes to obtain the competence of *L. plantarum* C8, which was named competence 1 (C1). It was stored for a long time in an ultra-low temperature refrigerator at −80°C for future use.

#### Electroporation of the auxiliary plasmid pLH01

The auxiliary plasmid pLH01 was introduced into *L. plantarum* C8 competence 1 (C1) via electroporation. Briefly, competence 1 was thawed on ice and mixed with less than 1 μg of pLH01 plasmid DNA in a total volume of under 1 μL. After incubating on ice for 10 min, the mixture was transferred to a pre-chilled 2 mm electroporation cuvette and left on ice for an additional 30 min. Electroporation was performed under the following conditions: 2 kV, 400 Ω, 4 ms, and 25 μF.

Immediately after electroporation, 900 μL of pre-warmed recovery medium (MRS broth supplemented with 3.423 g sucrose and 190.42 mg anhydrous MgCl₂ per 20 mL) was added. The mixture was incubated at 37°C for 3 h, then centrifuged at 4°C, 5,000 rpm for 3 min. The supernatant was discarded, and the cells were resuspended in 1 mL of antibiotic-free MRS broth. Transformants were selected on MRS agar plates containing 12.5 μg/mL chloramphenicol and incubated at 37°C for 24–48 h. Single colonies were picked, expanded in liquid culture for 12 h, and verified by PCR. Colonies showing correct amplification were confirmed as *L. plantarum* C8 successfully transformed with pLH01.

#### Induction expression and competent preparation of the auxiliary plasmid pLH01

The *L. plantarum* C8 strain containing the auxiliary plasmid pLH01 obtained in the previous step was inoculated into MRS Liquid medium containing antibiotics (chloramphenicol, 12.5 μg/mL), and shaken and cultured at 37°C until the mid-index stage to ensure the viability of the strain. Subsequently, add the induction peptide (MAGNSSNFIHKIKQIFTHR, 100 ng/mL) to the bacterial liquid to induce expression for 1–2 h. The induced bacterial liquid was prepared into competence according to the method described in “Preparation of *L. plantarum* C8 competence,” above. Finally, the competent state of *L. plantarum* C8 containing the auxiliary plasmid pLH01 was obtained and named competence 2 (C2).

#### Construction of recombinant strains

Recombinant strains were constructed by means of electrical transformation. The steps are as follows: the recombinant plasmid (Δ-pHSP02) was extracted according to the plasmid extraction method and was, respectively, introduced into *L. plantarum* C8 competence containing the auxiliary plasmid pLH01 along with the empty plasmid. The electrical transfer formation steps and conditions were the same as those described in “Electroporation of the auxiliary plasmid pLH01,” above. The electrical transfer results were tested by a resistant MRS solid medium containing chloramphenicol (12.5 μg/mL) and erythromycin (12.5 μg/mL). Monoclonal colonies were picked for bacterial liquid PCR. The PCR products were verified by 1% agarose gel electrophoresis. After the band position and size were in line with expectations, they were sent to Shanghai Shenggong Biotechnology Engineering Co., Ltd. for sequencing. It was finally confirmed that the recombinant strain was the *srtA* deletion mutant strain of *L. plantarum* C8, and the empty vector strain was the *L. plantarum* C8 strain containing the empty plasmids pHSP02 and pLH01.

#### Reverse-transcription quantitative PCR

The wild-type strains (WT), empty vector strains (EV), and the *srtA* knockout strains (KO) were activated overnight. RNA extraction was performed using fresh bacterial solutions. The extraction steps were in accordance with the HiPure Bacterial RNA Kit of Majorbio. The concentration and purity of the products were verified by an ultra-micro spectrophotometer. Using the RNA extracted above as the template, reverse transcription was performed with reference to the Novozyme HiScript III All-in-one RT SuperMix Perfect for qPCR kit. The reaction system is shown in Table 1.

**TABLE 1:** RNA reverse transcription reaction system

Reagent	Volume
5× All-in-one qRT SuperMix	4 μL
Enzyme mix	1 μL
Template RNA	1 pg–1 µg
RNase-free water	Up to 20 μL

Gently mix and then instantaneously centrifuge to make the liquid sink to the bottom. Reaction conditions: 37°C for 2 min; 55°C for 10 min; 85°C for 10 s, resulting in the production of cDNA. The primers required for the reverse transcription-quantitative PCR (RT-qPCR) are shown in Table 2. *srt*-F/R is the specific amplification primer for *srtA*, and rpoD-F/R is used as the internal reference. The primers were synthesized by Hangzhou Youkang Biotechnology Co., Ltd. The RT-qPCR steps were in accordance with the Novozyme Taq Pro Universal SYBR qPCR Master Mix manual. After the reaction was completed, the expression level of the target gene was analyzed according to the ΔΔCT method (24). The fluorescence quantitative program is set as follows: 95°C, 30 s; 95°C for 5 s, 60°C for 15 s, a total of 45 cycles.

**TABLE 2:** Primer design and reaction system for RT-qPCR

Primer name	Primer sequence (5′ to 3′)	Base number (bp)
*srt*-F	CGTAAACAAATTAGGAGGCGGGATTAATG	29
*srt*-R	ACCCCTAGAATGTAATAATTGTTAATATTTGTTATTAAAATGACTTGT	48
*rpoD*-F	AGACGTTTTCTTCCCGGTCC	20
*rpoD*-R	AAGATTTAGGGCGCGAACCA	20

### Scanning electron microscopy and transmission electron microscopy analysis

The ultrastructures of *L. plantarum* C8 WT strain, EV strain, and the *srtA* KO strains were observed by scanning electron microscopy (SEM) and transmission electron microscopy (TEM). Three bacteria in 2%–3% quantity of access with or without antibiotic MRS liquid medium (the addition of antibiotics from left to right was antibiotic-free, 12.5 μg/mL chloramphenicol, 12.5 μg/mL chloramphenicol + 12.5 µg/mL erythromycin.); WT was incubated at 37°C, while EV and KO were incubated overnight at 30°C and 200 rpm. The logarithmic phase cells were collected by centrifugation and washed with PBS. The SEM samples were fixed at 2.5% glutaraldehyde at 4°C for 12 h, subjected to gradient ethanol dehydration (30%–100%), critical point drying, gold spraying treatment, and finally imitated in Hitachi S3400N high vacuum mode. For the TEM analysis, samples were kept in 2.5% glutaraldehyde for 12 h at 4°C and washed three times with 0.1 M PBS (10 min, 4°C), followed by keeping in 1 % osmic acid for 2 h at room temperature. Then, specimens were dehydrated with ethanol (30%, 50%, 70%, and 90% ), 90% acetone, and 100% acetone. After that, the samples were treated with fresh Spurr’s resin and polymerized (37°C, 12 h, and 60°C, 72 h). The stained specimens were observed with TEM (Hitachi model H-7650).

### Determination of growth rate and acid production capacity of recombinant strains

To compare the growth kinetics and acid production capacity of WT, EV, and KO strains, three kinds of bacteria in 2%–3% quantity, respectively, in MRS liquid medium containing or without antibiotic (antibiotic-free, 12.5 μg/mL chloramphenicol, 12.5 μg/mL chloramphenicol + 12.5 µg/mL erythromycin) in the early growth. WT was left to stand at 37°C, and EV and KO were incubated in a rotary shaker overnight at 30°C, 200 rpm. When the bacterial cultures reached the logarithmic growth phase, the cells were harvested by centrifugation and washed three times with sterile PBS (0.01 M) (4°C, 5,000 r/min, 5 min). The concentration was adjusted to an OD_600_ of 1.0 ± 0.01, and 2% (vol/vol) of this suspension was then inoculated into antibiotic-free MRS broth and incubated at 37°C for 30 h. During this period, samples were taken every 2 h to measure OD₆₀₀ (Tecan Infinite M200 Pro) to plot the growth curve, and the pH of the culture medium (FE28-Meter) was measured simultaneously to evaluate the acid production capacity.

### Auto-aggregation and hydrophobicity assay

The WT, EV, and KO strains were inoculated at 2%–3% into MRS broth with or without antibiotics. The WT strain was grown statically at 37°C, while the EV and KO strains were cultured overnight at 30°C and 200 r/min. When the bacterial cultures reached the logarithmic growth phase, the cells were harvested by centrifugation and washed three times with sterile PBS (0.01 M) (4°C, 5,000 r/min, 5 min). The concentration was adjusted to an OD_600_ of 1.0 ± 0.01, and 2% (vol/vol) of this suspension was then inoculated into antibiotic-free MRS broth and incubated at 37°C. For the auto-aggregation assay, the initial absorbance (A₀) was recorded, and the suspension was left standing. Absorbance (A₁) was measured every 2 h for 12 h, and the auto-aggregation rate was calculated as


(1) $$Auto\text{-}aggregation\;(\%)=\left(1-\frac{A_{t}}{A_{0}}\right)\times 100.$$

For the surface hydrophobicity test, 3 mL of the bacterial suspension was mixed with hexane, xylene, or chloroform at a 3:1 ratio and incubated at 37°C for 10 min. After thorough mixing, samples were left at 37°C for 3 h, and the absorbance of the aqueous phase (B₁) was measured. Hydrophobicity was calculated as


(2) $$Hydrophobicity\;(\%)=\left(1-\frac{B_{1}}{B_{0}}\right)\times 100.$$

### Gastrointestinal tolerance of recombinant *Lactobacillus*

Bacterial cultures were prepared as described above, adjusted to OD_600_ = 1.0 ± 0.01, and aliquoted into sterile 1.5 mL microcentrifuge tubes (1 mL per tube). For each strain, three groups were set up: blank (no treatment), simulated gastric fluid (SGF), and simulated intestinal fluid (SIF). The blank group was serially diluted, and appropriate dilutions were plated on MRS agar without antibiotics. The SGF group involved resuspension in equal volumes of SGF (pH 2.0, 0.5% NaCl, 0.3% pepsin) and incubation at 37°C for 2 h, followed by washing and resuspension in sterile deionized water. The SIF group used the SGF-treated cells, washed and resuspended in simulated intestinal fluid (pH 8.0, 0.5% NaCl, 0.5% bile salts, 0.3% trypsin), and incubated for 3 h at 37°C. All samples were then diluted and plated under the same conditions as the blank group. Washing conditions throughout the experiment were 4°C, 5,000 rpm, 5 min. All absorbance readings were taken at OD₆₀₀ using a Tecan Infinite M200 Pro microplate reader.

### Cytotoxicity of recombinant *Lactobacillus* to Caco-2 cells

Caco-2 cells in good condition were seeded into 96-well plates and cultured at 37°C with 5% CO₂ for 24 h. After culturing *Lactobacillus* strains at 37°C, bacterial cells were harvested, washed with sterile PBS, and adjusted to concentrations of 1 × 10⁷, 1 × 10⁸, and 1 × 10⁹ CFU/mL. Cells were treated with 10 μL of bacterial suspensions, incubated for 2 h, followed by the addition of 10 μL CCK-8 reagent and further incubation for 4 h. Absorbance was measured at 450 nm using a microplate reader. Cell viability was calculated using the following formula:


$$Cell\;viability\;(\%)=\left[\frac{\left(A_{1}-A_{3}\right)}{\left(A_{2}-A_{3}\right)}\right]\times 100,$$

where A_1_ is the absorbance of the treatment group, A_2_ is the untreated control, and A_3_ is the blank (no bacteria and no cells).

### The influence of *srtA* deficiency on the biofilm formation ability

The WT, EV, and KO strains were inoculated into MRS Broth with or without antibiotics at a vaccination rate of 2%–3%. The WT strain was statically cultured at 37°C, while the EV and KO strains were incubated overnight at 30°C and 200 r/min. Once the training to the logarithmic phase is complete, collect cells, centrifuge (4°C, 5,000 r/min, 5 min), with 0.01 M PBS washing three times, and adjust for OD_600_ = 1.0 ± 0.01 mm. On 96-well polystyrene microplates, 200 μL of antibiotic-free MRS Broth was added to each well, followed by 20 μL of bacterial suspension. The wells containing only MRS broth were used as blank controls. Incubate at 37°C for 24 h. After incubation, gently wash three times with sterile PBS to remove planktonic cells. The biofilm was immobilized with 200 μL of 96% methanol for 15 min and air-dried. Add 200 μL of 0.1% crystal violet solution to each well and stain at room temperature for 40 min. Rinse with sterile water to remove excess stains and ensure the board is completely dry. Finally, 100 μL of 90% ethanol was added to each well to dissolve the bound dye, and the absorbance was measured at 595 nm.

### Adhesion of recombinant *Lactobacillus* to Caco-2 cells

Bacteria were grown, washed, and adjusted to OD_600_ = 1.0 ± 0.01. FITC was added to the suspensions to a final concentration of 0.1 mg/mL and incubated at 37°C in the dark for 30 min. Cells were then washed, and the initial fluorescence (A_0_) was recorded using a microplate reader (excitation 495 nm, emission 519 nm). Caco-2 cells were seeded in 6- and 12-well plates and cultured overnight to reach 70%–90% confluence. FITC-labeled bacteria were centrifuged, resuspended in DMEM, and added to wells (2 mL for 6-well, 1 mL for 12-well) for 2 h incubation in the dark. After removing non-adherent bacteria via washing, adhesion was visualized using a fluorescence microscope. For quantification, the 12-well plates were treated with trypsin, and the fluorescence intensity of adhered bacteria (A_1_) was measured. Adhesion rate was calculated as


$$Adhesion\;rate\;(\%)=\left(\frac{A_{1}}{A_{0}}\right)\times 100.$$

### Transcriptomic analysis

The WT, EV, and KO strains were streaked onto MRS agar plates with or without antibiotics and purified over three passages (WT strain: 37°C, EV strain and KO strain: 30°C). After the strains were purified, the single colonies were placed in MRS liquid medium at 37°C to grow to the logarithmic growth phase. The cultures were centrifuged, and the resulting pellets were transferred into sterile cryotubes (minimum pellet volume 0.1 mL or ≥0.1 g), flash-frozen in liquid nitrogen for 30 min, and stored at −80°C until shipment on dry ice for sequencing.

RNA sequencing and quality control were performed by Shanghai Majorbio Co., Ltd. Libraries were prepared using the TruSeqTM Stranded Total RNA Library Prep Kit, and sequencing was carried out on NovaSeqXPlus and DNBSEQ-T7 platforms. Raw data were filtered to remove adapters, duplicates, and low-quality reads, producing clean data. Reads were mapped to the *L. plantarum* C8 reference genome for downstream analysis. Library quality was assessed based on saturation, gene coverage, and read distribution across genomic regions. Gene expression was quantified using RSEM in FPKM units. Differentially expressed genes (DEGs) were identified using DESeq2_EBSeq (criteria: *P* adjust < 0.05 and |log_2_FC| ≥ 1). Functional annotation of DEGs was performed based on Gene Ontology (http://www.geneontology.org/) and KEGG (http://www.genome.jp/kegg/) databases.

### Statistical analysis

All statistical analyses were performed using one-way ANOVA in 26 SPSS software for significance analysis of variance, and multiple comparisons were performed using LSD and Duncan multiple comparisons. *P* < 0.05 was considered statistically significant. All values were expressed as mean ± standard deviation (X ± SD).

## RESULTS

### Construction of *L. plantarum* strains with *srtA* gene knock out

**Fig 1:**
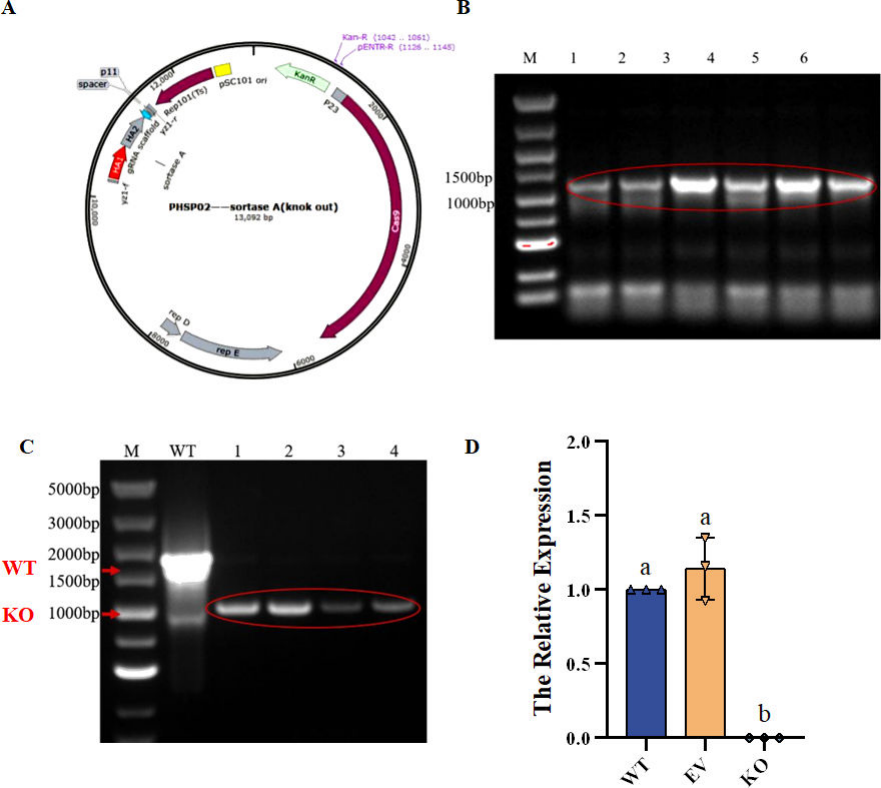
Recombinant knockout plasmid construction. (**A**) Knockout plasmid construction map; (**B**) verification of recombinant plasmid transformation. Lanes 1 to 5 were all picked single colonies; (**C**) PCR verification of recombinant knockout strain bacterial liquid; (**D**) the expression differences of *srtA* in different strains. Different letters indicate significant differences (*P* < 0.05).

In this study, the knockout plasmid targeting *srtA* was successfully constructed by homologous recombination of the linearized plasmid and the target fragment. The recombinant plasmid includes the upstream and downstream homologous arms (HA1, HA2) of *srtA*, sgRNA scaffold, and the P11 promoter carrying N20, as shown in Fig. 1A. After the recombinant plasmid was thermally transformed into *Escherichia coli* DH5α, monoclones were screened by resistance plates and PCR detection in planktonic bacteria was performed, as shown in Fig. 1B. 1% Agarose gel electrophoresis showed that a single and bright band (lanes 1–6) was amplified at about 1,200 bp, and the size of the band was consistent with expectations.

After the recombinant plasmid was introduced into the competent cells of lactic acid bacteria, the successfully surviving monoclonal strains were selected under the double antibiotic conditions for PCR detection in planktonic bacteria (i.e., non-aggregated cells). If *srtA* mutates successfully, the size of the product should be the sum of the bases of the two homologous arms, approximately 1,000 bp. If *srtA* fails to mutate successfully, a product of approximately 1,700 bp should be amplified. As shown in Fig. 1C, the results of 1% agarose gel electrophoresis indicated that compared with the wild strain (lanes: compared with WT), the recombinant strain (lanes 1–4) showed a base deletion of about 700 bp. The sequencing identification results also indicated that the *srtA* gene sequence was completely deleted, indicating that the *srtA* knockout was successful in *L. plantarum* C8, and the obtained mutant strain can be used for subsequent experiments.

The RT-qPCR results (Fig. 1D) showed that there was no significant difference in the expression level of *srtA* between the WT and EV strains, indicating that the introduction of the empty plasmid did not affect the normal expression of *srtA*. However, in the KO strain, the mRNA of *srtA* was not detected, and its expression level was missing. This result further proves that *srtA* has been successfully knocked out in the KO strain.

### SEM and TEM analysis of *L. plantarum* C8

**Fig 2:**
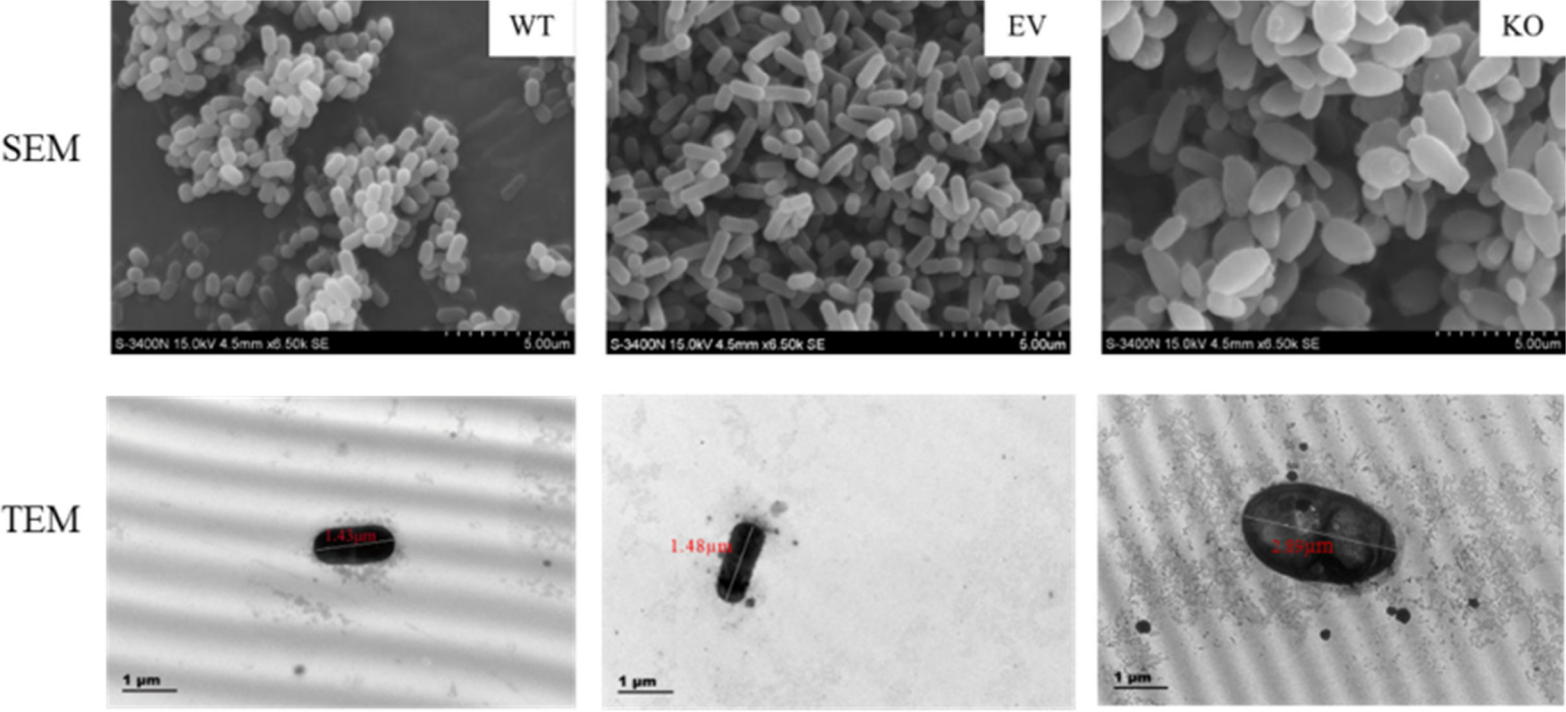
Phenotypic changes in strains before and after *srtA* deletion (SEM and TEM). It is divided into three groups: the wild-type strains (WT), empty vector strains (EV), and the *srtA* knockout strains (KO).

The morphology of *L. plantarum* C8 was detected by SEM and TEM, as shown in Fig. 2. The results of SEM showed that both the WT and EV strains were short rod-shaped strains with intact shapes and clear edges. WT aggregates more closely, while EV is slightly dispersed, indicating that the empty plasmid does not change the morphology of the strain, but may slightly affect the aggregation among bacteria. On the contrary, the KO strain showed obvious abnormalities—the cells were deformed, collapsed, rough, and unevenly aggregated. Similar results were also detected in the TEM analysis, which revealed that the rod shapes of the WT and the EV strains were regular, the membranes were intact, and the internal structures were uniform. However, the recombinant strains (KO group) were oval-shaped, internally disordered, with membrane damage, and were much larger in size (2.89 μm). The above results indicate that *Sortase A* plays an important role in stabilizing the cell wall structure of *L. plantarum* C8.

### Functional impact of *srtA* deletion on *L. plantarum* C8

**Fig 3:**
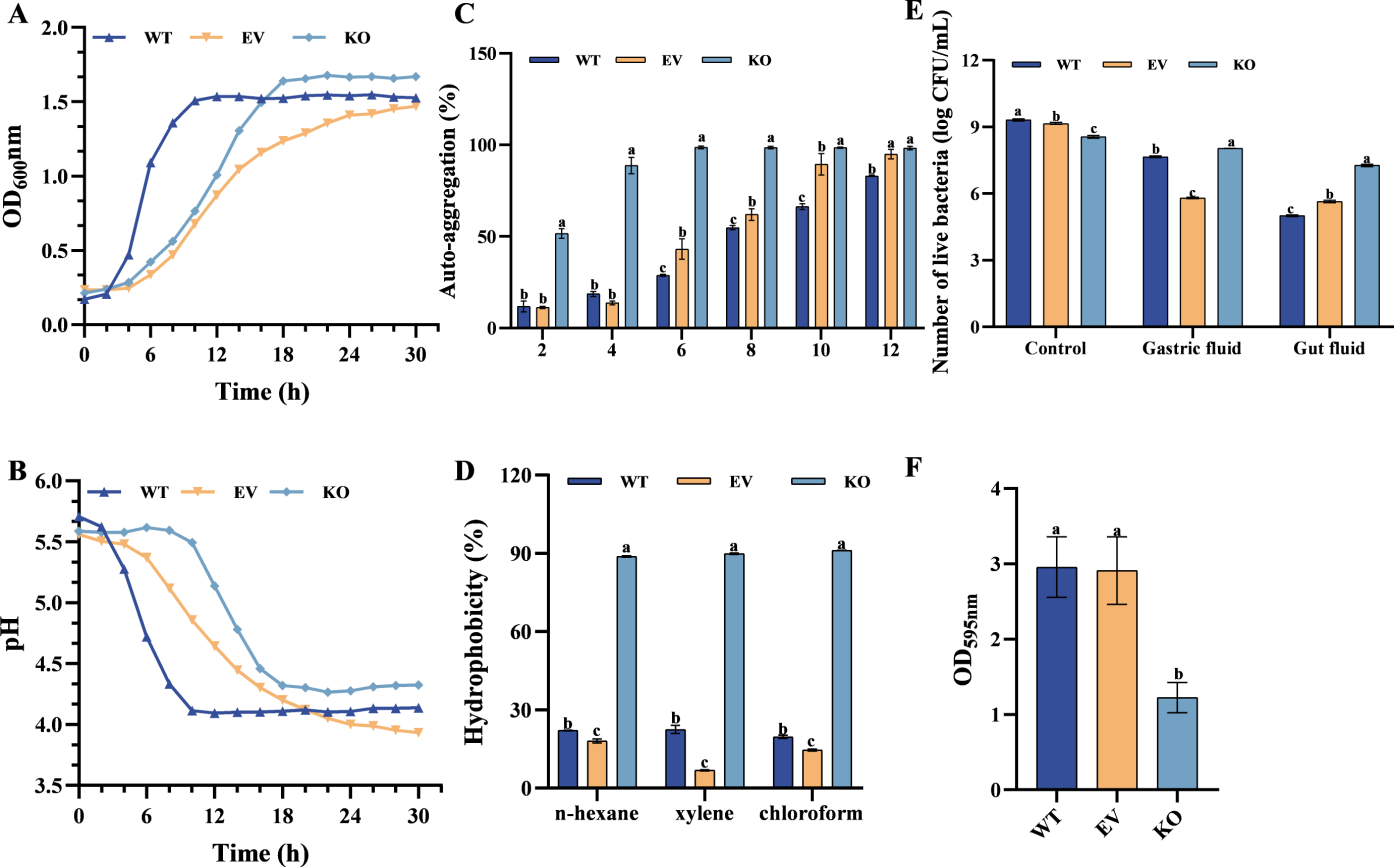
The influence of *srtA* deletion strains on the functional characteristics of the strains. (**A**) The influence of *srtA* deficiency on the growth rate of the strain; (**B**) the influence of *srtA* deficiency on the acid production capacity of strains; (**C**) change in the self-aggregation ability of strains over 12h; (**D**) hydrophobic capacity of strains against n-hexanene, xylene, chloroform; (**E**) effect of s*rtA* deletion on gastrointestinal tolerance of strains; (**F**) effect of *srtA* loss on biofilm formation. Different letters indicate significant differences (*P* < 0.05).

To elucidate the functional role of the *srtA* gene in *L. plantarum* C8, the effects of its deletion on growth dynamics, acid production, surface properties, gastrointestinal tolerance, cytotoxicity, and adhesion to intestinal epithelial cells were comprehensively evaluated. Growth curve analysis revealed distinct metabolic alterations in the *srtA* KO strain compared to WT and EV controls. WT entered exponential growth within 2 h, reaching the stationary phase at 10 h with rapid acidification (pH 4.1). EV exhibited delayed growth and reduced final cell density. KO initiated exponential growth at 10 h (Fig. 3A), achieving the stationary phase by 18 h, accompanied by gradual acidification (pH 4.4 at 18 h) (Fig. 3B). Analysis of cell surface characteristics demonstrated that *srtA* deletion significantly enhanced both autoaggregation and hydrophobicity. The KO strain achieved 98.68% autoaggregation by 6 h, maintaining this level through 12 h, while WT and EV reached 83.08% and 94.92%, respectively (Fig. 3C). Hydrophobicity assays confirmed elevated surface hydrophobicity in the KO strain (>88% across solvents) compared to WT and EV (Fig. 3D).

In Fig. 3E, gastrointestinal tolerance assays further revealed that *srtA* deletion increased survival under simulated gastric and intestinal conditions. After 2 h in artificial gastric fluid, KO strains retained higher viability (8.04 log CFU/mL) than EV strains (5.80 log CFU/mL) and maintained this advantage after intestinal fluid exposure. These results indicate enhanced acid and bile tolerance in the absence of *srtA*, which may facilitate survival in the gastrointestinal tract. The biofilm formation ability before and after the absence of *srtA* in *L. plantarum* C8 was also determined, and the results are shown in Fig. 3F. Both the wild strain and the empty strain showed high absorbance at 595 nm, indicating that the two strains had strong biofilm formation ability, and the introduction of the plasmid had no effect on the biofilm formation ability of *L. plantarum* C8. Meanwhile, the absorbance value of the recombinant strain at 595 nm was only 1.22, and the biofilm formation ability was significantly reduced. It is speculated that *srtA* may play an important role in maintaining the biofilm formation process of *L. plantarum*.

**Fig 4:**
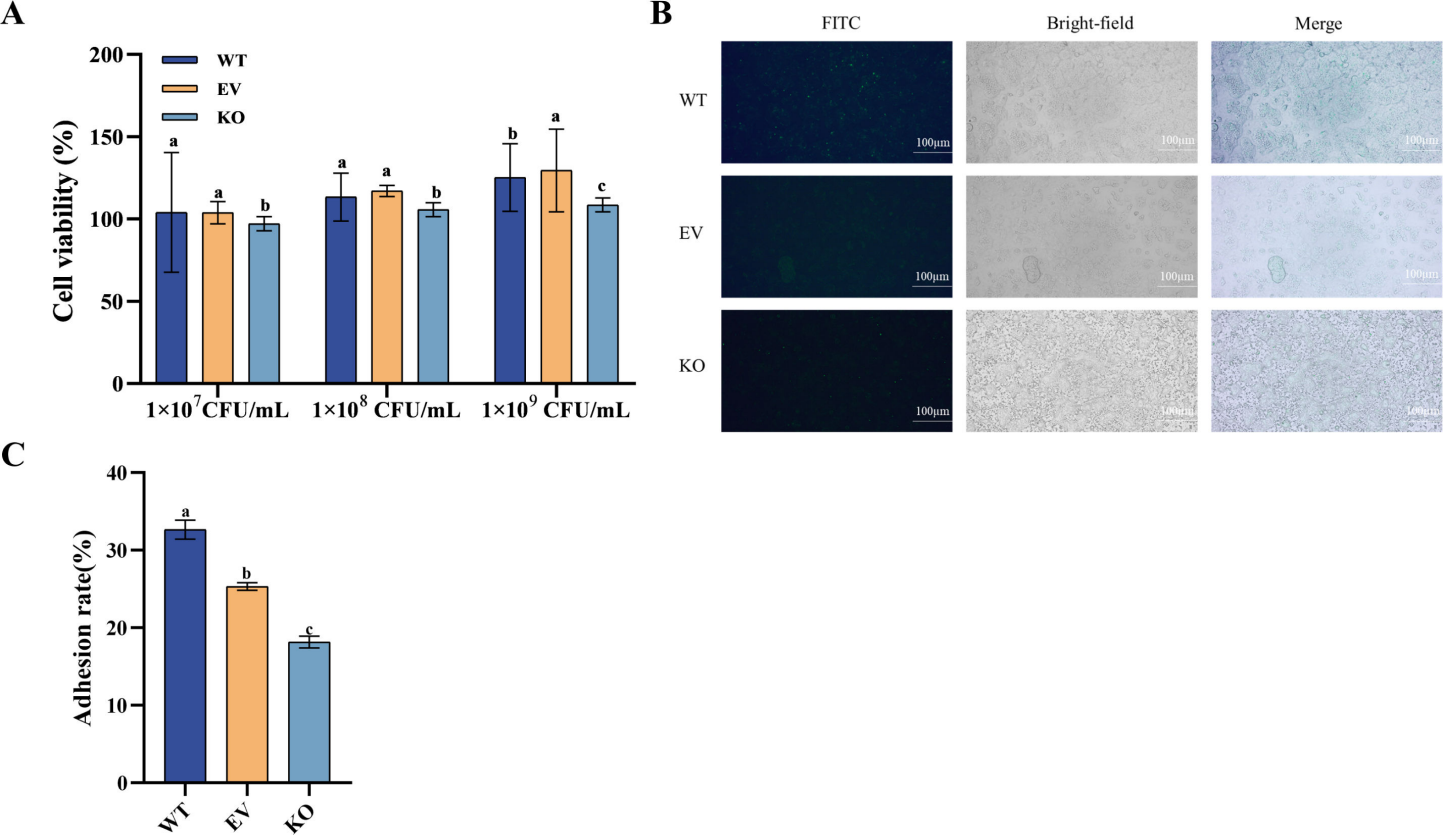
The Caco-2 cell experiment of s*rtA* deletion strains. (**A**) The toxic effect of *L. plantarum* C8 on Caco-2 cells; (**B**) adhesion of *L. plantarum* C8 to Caco-2 cells under the microscope (1, fluorescence image; 2, bright field plan; 3, superimposed image); (**C**) calculation of the adhesion rate of *L. plantarum* C8 to Caco-2 cells. Different letters indicate significant differences (*P* < 0.05).

To evaluate host interaction potential, Caco-2 cell models were used to assess cytotoxicity and adhesion. Co-culture experiments showed no significant cytotoxicity at bacterial concentrations up to 1 × 10⁹ CFU/mL, although KO strains induced a slight reduction in Caco-2 viability at the lowest dose (97.15%), with overall viability remaining above 105% in Fig. 4A. Fluorescence microscopy (Fig. 4B) revealed that WT strains exhibited the strongest adhesion, followed by EV and KO strains. KO samples displayed dispersed fluorescence signals with visible free bacteria in the culture medium, contrasting with the concentrated surface-associated fluorescence in WT and EV groups. Quantification of adhesion rates (Fig. 4C) confirmed this trend, with WT, EV, and KO strains showing adhesion rates of 32.66%, 25.31%, and 18.14%, respectively.

### Transcriptomic data of *L. plantarum* C8

**Fig 5:**
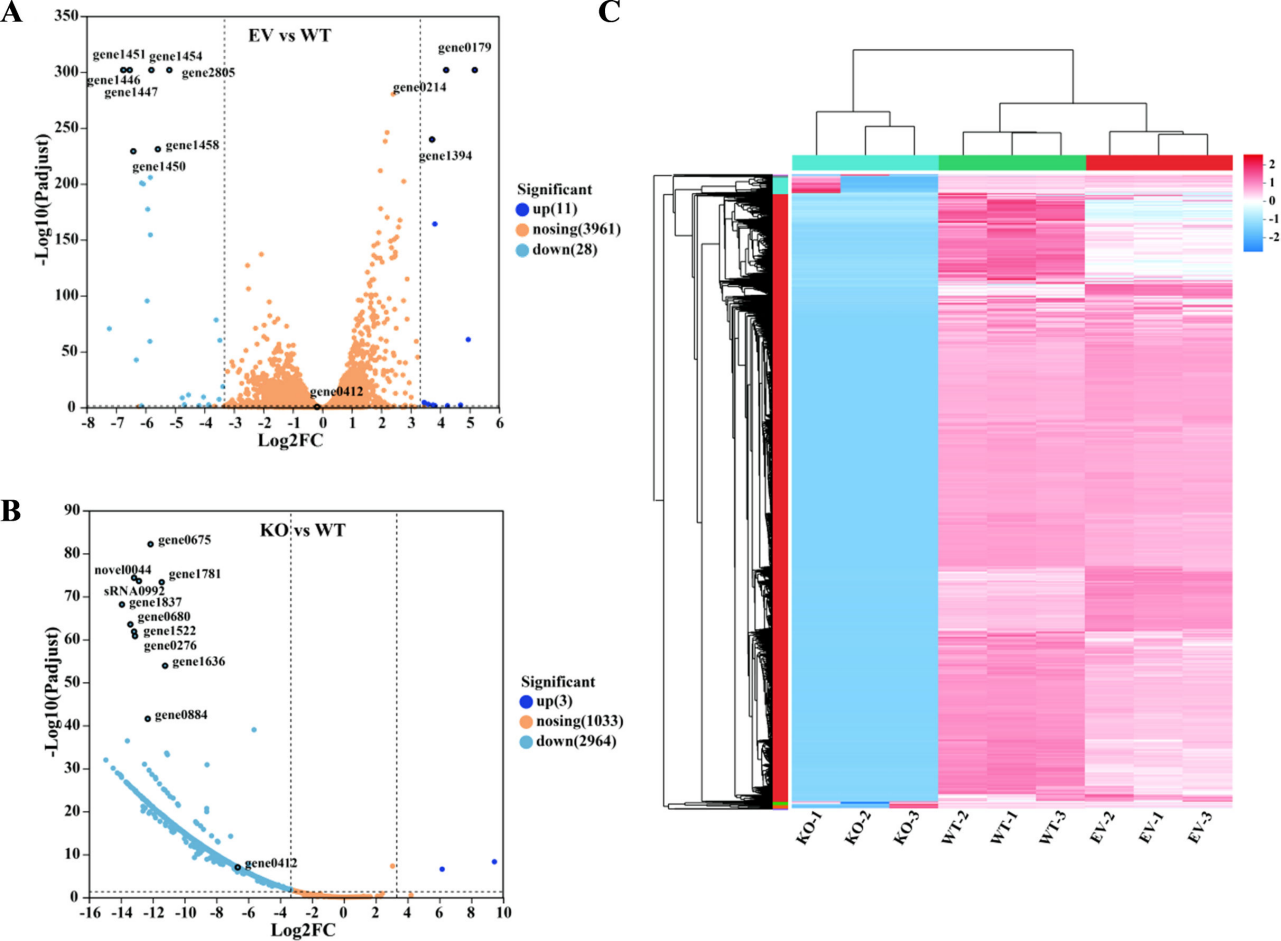
Differential gene expression analysis. (**A**) EV vs WT differential gene volcano plot; (**B**) volcano map of KO vs WT differential genes; (**C**) heat map of differential gene clustering.

Transcriptomic profiling of *L. plantarum* C8 WT, EV, and KO strains identified 2,967 DEGs in the KO strain (adjusted *P* < 0.05, |log_2_FC| ≥ 3.3), predominantly downregulated except for three hypothetical protein-coding genes (novel0153, novel0073, novel0129). Hierarchical clustering revealed distinct transcriptional divergence between the KO group and both WT/EV groups, demonstrating robust intra-group reproducibility across biological replicates (Fig. 5). EV DEGs centered on carbohydrate transport (phosphotransferase system, starch/sucrose metabolism), whereas KO DEGs emphasized environmental adaptation and energy metabolism pathways.

**Fig 6:**
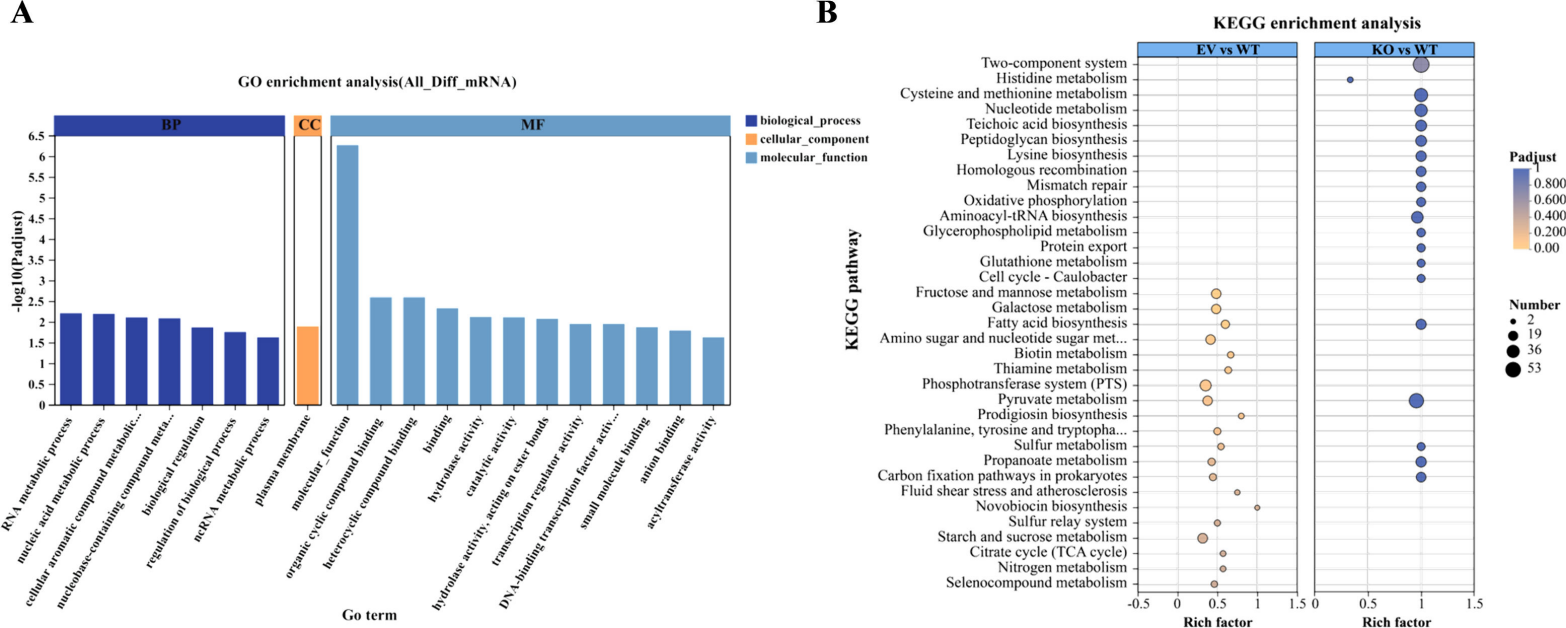
GO and KEGG pathway enrichment analysis. (**A**) Top 20 enriched GO terms of differentially expressed genes; (**B**) effects of *srtA* deletion on KEGG enriched pathways.

Gene Ontology (GO) enrichment analysis showed that the differentially expressed genes were mainly concentrated in the molecular function (MF) category, some were involved in biological processes (BP), and only a few belonged to cellular components (CC) (Fig. 6A). At the functional level, MF items related to metabolic enzyme activities, DNA-binding transcriptional regulatory factor activities, and small molecule binding were significantly enriched. In terms of BP, pathways such as transcriptional regulation, RNA, and nucleic acid metabolism are enriched. It is worth noting that the only significantly enriched CC term was the plasma membrane (GO: 0005886), which was consistent with the changes in cell surface characteristics (such as enhanced auto-aggregation and hydrophobicity). KEGG pathway analysis highlighted metabolic reprogramming in the KO strain, with enriched pathways including two-component signal transduction, pyruvate metabolism, and amino sugar/nucleotide sugar metabolism. Among them, the two-component system is an important signal transduction system that is widely involved in environmental perception and stress response (25, 26). Comparison between the KO and EV groups further highlighted distinct transcriptomic responses (Fig. 6B).

Targeted analysis of cell wall biosynthesis identified 26 downregulated peptidoglycan-related genes in the KO strain, including Mur enzyme family members (*murA–murF*). Among 20 adhesion-associated DEGs, Sec secretion system components (*secA, secY, secE, secG*) and surface interaction genes (gene2222, gene2582) exhibited marked downregulation. Biofilm-related transcriptional changes included reduced expression of 15 DEGs linked to extracellular polysaccharide biosynthesis (*glgC, glgA, glgP*) and QS (*luxS*/gene0668), with all biofilm-associated genes showing significant downregulation in the KO strain (Tables 3 to 5). The EV group displayed minimal transcriptional perturbations, confirming the specific effects of the *srtA* deletion strain. In Fig. S1, four genes related to biofilms, adhesion, and cell walls were selected for further verification, and the results were consistent with expectations. The corresponding RT-qPCR primer design is presented in Table S1.

**TABLE 3:** Differential genes associated with cell wall synthesis^*a*^

Gene ID	Gene name	Functional description	Difference change	
			FC (KO vs WT)	FC (EV vs WT)
gene1585	*tarI*	D-ribitol-5-phosphate cytidylyltransferase	1.14 × 10^−3^	1.02
gene0663	*vanX*	Zinc D-Ala-D-Ala dipeptidase	3.10 × 10^−2^	2.00
gene1062/2750	*dacC*	Serine-typeD-Ala-D-Ala carboxypeptidase (penicillin-binding protein 5/6)	3.24 × 10^−3^/5.33 × 10^−4^	2.62/0.50
gene2027	*ddl*	D-alanine-D-alanine ligase	3.11 × 10^−4^	1.52
gene1899	*mraY*	Phospho-N-acetylmuramoyl-pentapeptide-transferase	5.95 × 10^−4^	1.26
gene0408/2040	*murA*	UDP-N-acetylglucosamine 1-carboxyvinyltransferase	1.09 × 10^−3^/1.14 × 10^−3^	0.87/0.35
gene0702	*murB*	UDP-N-acetylmuramate dehydrogenase	2.09 × 10^−3^	1.06
gene1258	*murC*	UDP-N-acetylmuramate–alanine ligase	5.56 × 10^−4^	1.02
gene1898	*murD*	UDP-N-acetylmuramoylalanine–D-glutamate ligase	1.28 × 10^−3^	0.38
gene0851	*murE*	UDP-N-acetylmuramoyl-L-alanyl-D-glutamate–2,6-diaminopimelate ligase	9.37 × 10^−4^	1.17
gene0416	*murF*	UDP-N-acetylmuramoyl-tripeptide–D-alanyl-D-alanine ligase	9.88 × 10^−4^	1.25
gene1897	*murG*	UDP-N-acetylglucosamine–N-acetylmuramyl-(pentapeptide) pyrophosphoryl-undecaprenol N-acetylglucosamine transferase	4.74 × 10^−3^	0.61
gene1961	*murI*	Glutamate racemase	4.81 × 10^−3^	0.70
gene2056/2057	*murT/gatD*	Lipid IIisoglutaminyl synthase (glutamine-hydrolyzing)	1.89 × 10^−3^/1.80 × 10^−3^	1.34/1.24
gene1732	*amiABC*	N-acetylmuramoyl-L-alanine amidase	1.38 × 10^−3^	0.93
gene1211/1745	*dltC*	D-alanine–poly(phosphoribitol) ligase subunit 2	2.29 × 10^−3^/9.38 × 10^−3^	1.82/1.67
gene0480	*tagA*	N-acetylglucosaminyldiphosphoundecaprenolN-acetyl-beta-D-mannosaminyl transferase	2.30 × 10^−3^	0.77
gene0985	*acm*	Lysozyme	5.92 × 10^−3^	1.77
gene0626	*wecA*	UDP-GlcNAc:undecaprenyl-phosphate/decaprenyl-phosphate GlcNAc-1-phosphate transferase	1.42 × 10^−3^	1.00
gene3133	*jag*	SpoIIIJ-associated protein	1.98 × 10^−3^	0.82
gene2908	*bacA*	Undecaprenyl-diphosphatase	1.25 × 10^−3^	0.97
gene0391	*glmU*	Bifunctional UDP-N-acetylglucosamine pyrophosphorylase/glucosamine-1-phosphate N-acetyltransferase	3.94 × 10^−4^	1.06

^*a*^ FC, fold change.

**TABLE 4:** Differential genes related to adhesion^*a*^

Gene ID	Gene name	Functional description	Difference change	
			FC (KO vs WT)	FC (EV vs WT)
gene0412	*srtA*	Sortase A	9.90 × 10^−3^	0.89
gene2222	*WP_301698875.1*	SpaA isopeptide-forming pilin-related protein	1.32 × 10^−2^	0.98
gene2582	*EFK30052.1*	Zinc/manganese transport system substrate-binding protein	3.62 × 10^−2^	0.93
gene0939	*mntC*	Manganese transport system substrate-binding protein	2.83 × 10^−2^	2.01
gene1744	*dltD*	D-alanine transfer protein	1.66 × 10^−4^	1.73
gene0683/1668	*eno*	Enolase 1/2/3	3.19 × 10^−5^/9.33 × 10^−2^	0.80/0.60
gene1051	*oppD*	Oligopeptide transport system ATP-binding protein	3.43 × 10^−4^	1.16
gene1550	*lspA*	Signal peptidase II	4.81 × 10^−2^	0.72
gene1416	*ffh*	Signal recognition particle subunit SRP54	1.09 × 10^−3^	1.61
gene1414	*ftsY*	Fused signal recognition particle receptor	1.32 × 10^−3^	1.48
gene0632	*secA*	Preprotein translocase subunit SecA	4.03 × 10^−4^	1.07
gene0520	*secE*	Preprotein translocase subunit SecE	7.74 × 10^−3^	1.17
gene0686	*secG*	Preprotein translocase subunit SecG	1.48 × 10^−3^	1.10
gene0905	*secY*	Preprotein translocase subunit SecY	7.90 × 10^−5^	2.08
gene1973	*yajC*	Preprotein translocase subunit YajC	1.05 × 10^−3^	2.11
gene3136/1335	*yidC*	YidC/Oxa1 family membrane protein insertase	1.26 × 10^−4^/1.23 × 10^−3^	1.24/0.92
gene0677/1162	*clpP*	ATP-dependent Clp protease, protease subunit	2.92 × 10^−4^/4.25×10^−4^	0.75/0.22

^*a*^ FC, fold change.

**TABLE 5:** Differential genes related to the formation of biofilms^*a*^

Gene ID	Gene name	Functional description	Difference change	
			FC (KO vs WT)	FC (EV vs WT)
gene0020	*glgC*	Glucose-1-phosphate adenylyltransferase	7.16 × 10^−2^	0.81
gene0021	*glgA*	Starch synthase	6.24 × 10^−2^	1.02
gene0022	*glgP*	Glycogen phosphorylase	2.17 × 10^−2^	0.95
gene0210	*cysE*	Serine O-acetyltransferase	2.01 × 10^−2^	0.18
gene0668	*luxS*	S-ribosylhomocysteinelyase	7.11 × 10^−4^	1.10
gene0678	*rpoN*	RNA polymerase sigma-54 factor	7.93 × 10^−3^	1.08
gene0760/2572/3021	*crr*	Sugar PTS system EIIA component	0.12/0.25/1.61 × 10^−2^	0.76/1.58/0.90
gene0998	*wecB*	UDP-N-acetylglucosamine 2-epimerase (non-hydrolyzing)	1.86 × 10^−3^	0.58
gene0626	*wecA*	UDP-GlcNAc:undecaprenyl-phosphate/decaprenyl-phosphate GlcNAc-1-phosphate transferase	1.42 × 10^−3^	1.00
gene1314/1855/2321	*pgaC*	Poly-beta-1,6-N-acetyl-D-glucosamine synthase	1.33 × 10^−2^/3.63 × 10^−3^/1.09 × 10^−2^	0.80/2.07/1.36
gene1430	*trpE*	Anthranilate synthase component I	6.09 × 10^−2^	1.21

^*a*^ FC, fold change.

## DISCUSSION

*L. plantarum*, as a probiotic, its health-promoting effect largely depends on its colonization ability in the host body, among which intestinal adhesion is one of the key factors (27, 28). This process is tightly regulated by strain-specific surface proteins (29), many of which are anchored to the cell wall via sortase enzymes. Among them, SrtA functions as a membrane-anchored transpeptidase responsible for covalently linking LPXTG-motif-containing proteins to the peptidoglycan matrix (30, 31). In *Listeria monocytogenes*, the inactivation of the *srtA* gene inhibits the anchoring of surface proteins. The deletion of the *Staphylococcus aureus srtA* gene reduces the quantity of surface leucine, pilus, amino acid-containing, tripeptide, and glycine (LEXTG) proteins, affecting the anchoring of proteins and the formation and adhesion rate of biofilms. However, in non-model strains, such as *Lactobacillus plantarum* C8, this study found that although the phenotypic effects were similar, the transcriptional changes in *L. plantarum* C8 were more significant, which was attributed to the unique regulatory complexity of this strain.

In this study, a CRISPR/Cas9-based *srtA* knockout mutant of *L. plantarum* C8 was constructed and validated by RT-qPCR, revealing the core role of *srtA* in coordinating the integrity of the cell wall, the biofilm QS network, and adhesion function. Deletion of the *srtA* gene in *L. plantarum* C8 resulted in extensive phenotypic and transcriptomic changes, collectively underscoring the multifaceted role of *srtA* in maintaining probiotic functionality. Functionally, *srtA*-deficient strains exhibited markedly enhanced environmental adaptability, including significantly improved autoaggregation, surface hydrophobicity, and gastrointestinal fluid tolerance (32–35). These traits are critical for bacterial survival under host-induced stress, such as exposure to bile salts, acidic pH, and digestive enzymes. Enhanced auto-aggregation and hydrophobicity promote the formation of stable cellular clusters, which facilitate transient colonization and resilience within the unfavorable gut environment.

However, these adaptive advantages came at the cost of key probiotic functions. The *srtA* deletion strain showed impaired acid production and severely reduced adhesion to Caco-2 intestinal epithelial cells—two hallmark features of effective probiotic strains. Adhesion deficiency was further accompanied by a significant loss in biofilm formation capacity, indicating that cell–host interaction is critically dependent on *srtA*-anchored surface proteins. SrtA is responsible for anchoring LPXTG-containing proteins to the peptidoglycan layer; its absence may lead to the loss of surface-associated hydrophilic proteins, thereby increasing the exposure of underlying hydrophobic membrane components. To understand the mechanistic basis behind these phenotypic alterations, we examined both structural and transcriptional responses to *srtA* deletion. Morphological observations using SEM and TEM revealed significant structural abnormalities in the knockout strain. Cells exhibited severe deformation of the bacteria, significant expansion, dissolution, and shedding of membrane analogs—hallmarks of defective peptidoglycan synthesis.

These visual observations were supported by transcriptomic data showing downregulation of several key genes in the murA-murF family, which are crucial for peptidoglycan biosynthesis (28, 36–39). The disruption of these pathways strongly implicates *srtA* in maintaining cell wall homeostasis, likely through feedback mechanisms involving cell envelope stress responses. Not only that, the absence of *srtA* inhibits the formation of biofilms by simultaneously disrupting the QS and EPS biosynthesis pathways. The significant downregulation of *luxS* indicates potential impairment of AI-2-mediated intercellular communication, which plays a critical role in coordinating biofilm development in Gram-positive bacteria. Concurrently, decreased expression of *glg* family genes may reduce the synthesis of extracellular polysaccharides, which are essential for maintaining biofilm structure and promoting cell aggregation. This finding suggests a potential mechanism for the impaired biofilm phenotype observed in phenotypic assays and indicates that the influence of *srtA* may extend to the transcriptional regulation of processes critical for adhesion and environmental adaptation.

The unusually large number of differentially expressed genes observed following *srtA* deletion likely reflects a cascading cell-envelope stress response rather than a direct, gene-by-gene regulatory role of *srtA*. Loss of *srtA* prevents proper covalent anchoring of LPXTG-motif surface proteins to peptidoglycan, leading to mislocalization or secretion failure of multiple surface adhesions and enzymes. Such mislocalization can perturb cell envelope integrity and surface charge, provoking envelope stress sensors and remodeling pathways. As part of this response, bacteria commonly adjust expression of cell wall biosynthesis genes (e.g., Mur family), secretion pathway components (Sec machinery), and signal transduction systems to re-establish envelope homeostasis. Consequently, two-component systems and QS circuits—which monitor extracellular cues and coordinate population-level behaviors—are secondarily affected, resulting in broad transcriptional reprogramming. In short, *srtA* loss appears to trigger an indirect but global transcriptional adaptation aimed at mitigating envelope dysfunction and reallocating metabolic resources, which manifests as large-scale up- and down-regulation across diverse functional categories.

Transcriptome analysis further revealed that the absence of *srtA* triggered extensive transcriptional reprogramming, confirming that *srtA* is the hub of surface function. GO enrichment analysis identified a suite of downregulated genes involved in “cell adhesion,” “cell surface,” and “extracellular region” functions. Notably, genes encoding LPXTG-motif proteins and components of the Sec secretion system (s*ecA, secY, secE, secG*) were significantly suppressed (40–43). As the Sec pathway is central to the export and membrane localization of surface proteins in Gram-positive bacteria, its repression likely results in deficient protein translocation and reduced cell surface functionality. These molecular changes align well with the observed reductions in adhesion and biofilm formation.

Furthermore, KEGG pathway enrichment analysis revealed that DEGs in the *srtA* knockout strain were predominantly associated with stress response and signal transduction pathways—particularly the two-component system and QS. These systems are essential for environmental sensing and adaptive regulation of cell surface composition and membrane dynamics (44). Their downregulation suggests that *srtA* loss not only disrupts structural integrity but also perturbs regulatory networks responsible for environmental adaptation and host interaction.

Taken together, deletion of *srtA* initiates a cascading effect that spans structural, functional, and regulatory domains. The absence of *srtA*-mediated surface protein anchoring compromises peptidoglycan synthesis, damages cell envelope integrity, impairs adhesion systems, and activates a global transcriptomic response aimed at mitigating environmental stress. These findings position *srtA* as a central integrator of cell wall architecture, signal transduction, and probiotic functionality, highlighting its indispensable role in balancing host colonization with environmental persistence. Its deletion initiates a cascading failure across three interdependent tiers: structural collapse due to disabled surface protein anchoring, directly compromising specific adhesion; transcriptional diversion toward compensatory survival traits like auto-aggregation and hydrophobicity; QS disorder, the biofilm structure disintegrates (the physical barrier is lost), and the survival rate of bacteria decreases under the pressure of gastric acid, bile, etc.

This triad of defects—structural, functional, and regulatory—collectively demonstrates that SrtA transcends its canonical role as a cell wall enzyme. Rather, it functions as a master sentinel, synchronizing cell envelope integrity, signal transduction, and adhesive efficacy to maintain ecological competence.

### Conclusion

This study establishes that *sortase A (srtA*) governs *L. plantarum* C8 adhesion through a cell wall-centric regulatory triad: peptidoglycan integrity, biofilm-QS coordination, and surface functionality. CRISPR/Cas9-mediated *srtA* deletion triggered a series of cascading defects: the disruption of Mur enzyme-dependent peptidoglycan synthesis was associated with cell wall instability, likely compromising adhesion anchor sites. Concurrently, the suppression of *luxS* (involved in AI-2 signaling) and *glgC/A/P* (involved in EPS biosynthesis) was linked to impaired bacterial communication and biofilm formation; compensatory upregulation of hydrophobicity/aggregation genes enhanced gastrointestinal tolerance but irreversibly impaired host-specific adhesion. Transcriptomic and functional data confirm srtA as a non-redundant integrator of cell envelope homeostasis and probiotic efficacy. These mechanistic insights redefine srtA’s role beyond enzymatic anchoring, positioning it as a master regulator of probiotic resilience in gastrointestinal niches.

## Data Availability

The RNA sequencing data generated in this study have been deposited in the NCBI Sequence Read Archive (SRA) under the accession number PRJNA1404891.

## SUPPLEMENTAL MATERIAL

Supplemental material aem.00029-26-s0001.docx Fig. S1 and Table S1.

ASM does not own the copyrights to Supplemental Material that may be linked to, or accessed through, an article. The authors have granted ASM a non-exclusive, world-wide license to publish the Supplemental Material files. Please contact the corresponding author directly for reuse.
