# Supplementary material for: Sortase A regulates cell wall integrity, quorum sensing, and biofilm formation to modulate adhesion properties in Lactiplantibacillus plantarum C8
Source: Appl Environ Microbiol. 2026 Feb 25;92(3):e00029-26. doi: 10.1128/aem.00029-26 (PMC12997751; doi:10.1128/aem.00029-26)
Supplement: Supplemental material — Fig. S1 and Table S1. [file aem.00029-26-s0001.docx]

**Cell wall related biofilm-quorum sensing regulation mechanism of sortase A in the adhesion properties of *Lactiplantibacillus plantarum***

**
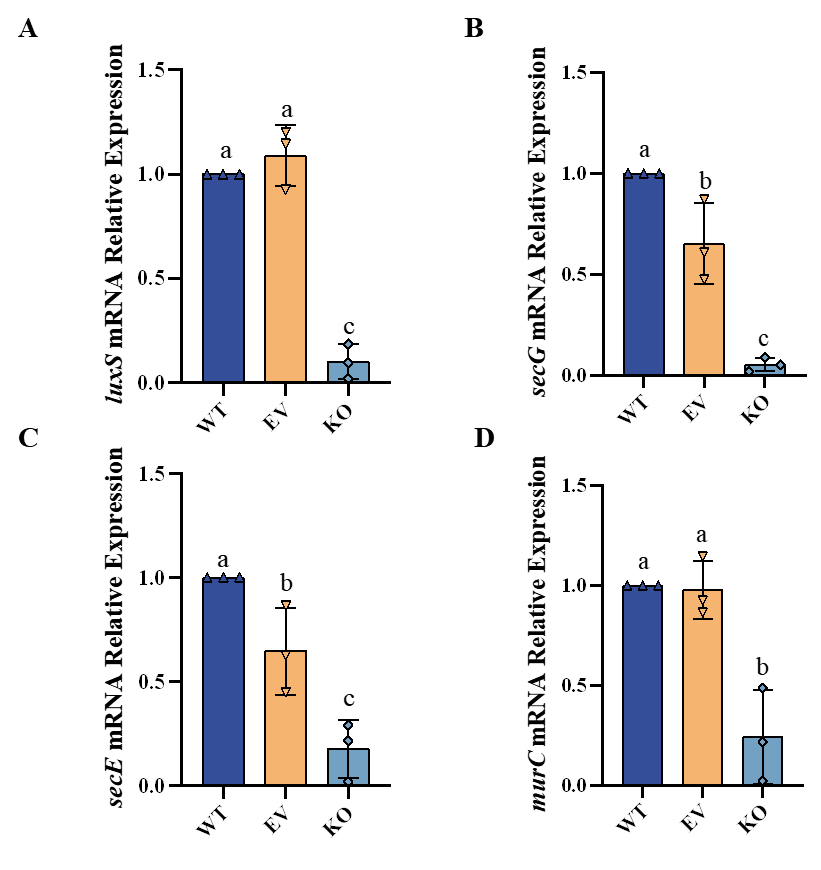
**

**Supplementary Fig. S1.** Analysis of the relative mRNA expression levels of *luxS*, *secG*, *secE*, and *murC* genes in different strains. (A) The relative expression levels of the *luxS* gene in WT, EV, and KO strains (B) Relative expression levels of the *secG* gene in different strains; (C) Relative expression levels of the *secE* gene in different strains; (D) Relative expression levels of the *murC* gene in different strains. Different letters indicate significant differences (p < 0.05).

**Supplementary Table S1** Primer design for RT-qPCR.

| Primer name | Primer sequence (5' to 3') | Base number/bp |
| --- | --- | --- |
| *secG*-F1 | TTGTATAATTTATTGTTAACGTTAATACTGGT | 32 |
| *secG*-R1 | TTACTTCGATGAGTACCATGCTAGAGC | 27 |
| *luxS*-F1 | CAACCTAACAAGACCGCTAT | 20 |
| *luxS*-R1 | AACCAGTCCGACAACCAA | 18 |
| *murC*-F1 | GAAGCTGATGAGTATCGAAGCGC | 23 |
| *murC*-R1 | CAATCAGTCGCTCCCAATCTTG | 22 |
| *secE*-F1 | ATGCGCTCATTTAAATTTTTTGG | 23 |
| *secE*-R1 | TTAATGCCAGCTTGTTAACATTTGT | 25 |
